# Sex Differences in Fish Oil and Olanzapine Effects on Gut Microbiota in Diet-Induced Obese Mice

**DOI:** 10.3390/nu14020349

**Published:** 2022-01-14

**Authors:** Mostafa M. Abbas, Paul Soto, Latha Ramalingam, Yasser El-Manzalawy, Halima Bensmail, Naima Moustaid-Moussa

**Affiliations:** 1 Department of Translational Data Science and Informatics, Geisinger, Danville, PA 17822, USA; mmhamza@geisinger.edu (M.M.A.); yelmanzalawi@geisinger.edu (Y.E.-M.); 2 Qatar Computing Research Institute, Hamad Bin Khalifa University, Doha 5825, Qatar; 3 Department of Nutritional Sciences, Obesity Research Institute, Texas Tech University, Lubbock, TX 79409, USA; soto1@lsu.edu (P.S.); lramalin@syr.edu (L.R.); 4 Pennington Biomedical Research Center, Louisiana State University, Baton Rouge, LA 70803, USA; 5 Department of Psychology, Louisiana State University, Baton Rouge, LA 70803, USA; 6 Department of Nutrition and Food Studies, Syracuse University, Syracuse, NY 13210, USA

**Keywords:** second-generation antipsychotic (SGA), obesity, fish oil

## Abstract

Children are prescribed second-generation antipsychotic (SGA) medications, such as olanzapine (OLZ) for FDA-approved and “off-label” indications. The long-term impact of early-life SGA medication exposure is unclear. Olanzapine and other SGA medications are known to cause excessive weight gain in young and adult patients, suggesting the possibility of long-term complications associated with the use of these drugs, such as obesity, diabetes, and heart disease. Further, the weight gain effects of OLZ have previously been shown to depend on the presence of gut bacteria and treatment with OLZ, which shifts gut bacteria toward an “obesogenic” profile. The purpose of the current study was to evaluate changes in gut bacteria in adult mice following early life treatment with OLZ and being fed either a high-fat diet or a high-fat diet supplemented with fish oil, which has previously been shown to counteract gut dysbiosis, weight gain, and inflammation produced by a high-fat diet. Female and male C57Bl/6J mice were fed a high fat diet without (HF) or with the supplementation of fish oil (HF-FO) and treated with OLZ from postnatal day (PND) 37–65 resulting in four groups of mice: mice fed a HF diet and treated with OLZ (HF-OLZ), mice fed a HF diet and treated with vehicle (HF), mice fed a HF-FO diet and treated with OLZ (HF-FO-OLZ), and mice fed a HF-FO diet and treated with vehicle (HF-FO). Following euthanasia at approximately 164 days of age, we determined changes in gut bacteria populations and serum LPS binding protein, an established marker of gut inflammation and dysbiosis. Our results showed that male HF-FO and HF-FO-OLZ mice had lower body weights, at sacrifice, compared to the HF group, with a comparable body weight across groups in female mice. HF-FO and HF-FO-OLZ male groups also exhibited lower serum LPS binding protein levels compared to the HF group, with no differences across groups in female mice. Gut microbiota profiles were also different among the four groups; the Bacteroidetes-to-Firmicutes (B/F) ratio had the lowest value of 0.51 in the HF group compared to 0.6 in HF-OLZ, 0.9 in HF-FO, and 1.1 in HF-FO-OLZ, with no differences in female mice. In conclusion, FO reduced dietary obesity and its associated inflammation and increased the B/F ratio in male mice but did not benefit the female mice. Although the weight lowering effects of OLZ were unexpected, FO effects persisted in the presence of olanzapine, demonstrating its potential protective effects in male subjects using antipsychotic drugs.

## 1. Introduction

Second-generation antipsychotics (SGAs) are an effective treatment option for adults suffering with psychiatric disorders and children suffering with schizophrenia and bipolar I disorder [1,2,3]. SGA medications are often prescribed for “off-label” indications [3] and are associated with rapid weight gain [4] and metabolic dysfunction, including obesity [5]. Olanzapine (OLZ) is one of the most commonly prescribed antipsychotics in children [3] and treatment with this drug has been shown to cause increased weight gain and metabolic syndrome [6,7]. However, long-term health implications associated with early-life exposure to SGA in terms of metabolic dysfunction remain unclear.

Various bioactives are known to prevent or reduce obesity. One of the bioactives of interest to us is fish oil (FO), which has beneficial effects in reducing obesity and related metabolic diseases in non-primate animals [8,9,10,11]. Fish oil contains n-3 long chain polyunsaturated fatty acids (n-3 PUFAs), which include α-linolenic acid, eicosapentaenoic acid (EPA), and docosahexaenoic acid (DHA). Our lab has previously published the beneficial effects of FO in reducing obesity, in part by reducing inflammation in white adipose tissue and serum [9,12]. However, whether FO can counteract weight gain due to SGA treatment has not been reported to our knowledge.

Numerous studies suggest that diet-induced obesity and insulin resistance are associated with alteration of the gut microbiota composition in mice and humans [13,14,15,16,17,18,19]. Specifically, at the phylum level, the ratio of Bacteroidetes-to-Firmicutes (B/F ratio) is decreased in individuals suffering from obesity compared to lean individuals [20,21,22]. Further, at the class level, Gram-negative *Bacteroidia* is decreased while Gram-positive *Clostridia* is increased in obese rodents compared to lean ones [23]. Moreover, Gram-negative *Akkermansia* at the genus level is negatively correlated with glucose intolerance [24,25]. Interestingly, OLZ appears to alter the composition of gut bacteria by increasing the relative abundance of “obesogenic” bacteria, in the absence of which OLZ does not produce increases in weight [26]. Additionally, studies have demonstrated that the effects of antipsychotics on gut microbiome are similar to individuals suffering from obesity (decreased B/F ratio) [27,28,29,30].

In this study, we evaluated the effects of FO and OLZ, independently and in combination, on markers of inflammation and adiposity, and determined potential associations between previously established anti-inflammatory and weight reducing effects of FO and gut microbial composition [31]. Accordingly, we hypothesized that FO might counteract antipsychotic-associated metabolic dysfunctions such as obesity, inflammation, and gut dysbiosis.

We report here that FO reduced obesity-associated inflammation. This is the first report of FO and OLZ’s differential effects on gut microbiome. Surprisingly, OLZ exhibited beneficial metabolic effects such as FO and did not counteract protective effects of FO. FO increased B/F ratio in male mice, and these effects persisted in the presence of OLZ, warranting further studies in both animal and human subjects using antipsychotic drugs.

## 2. Materials and Methods

### 2.1. Animals and Diet Procedure

All experimental procedures and animal care were approved and performed in accordance with the Texas Tech University Laboratory Animal Care under permit number 15007. Mice were randomized into four groups of mice (*n* = 8 per group): HF (HF), HF with olanzapine (HF-OLZ), HF with fish oil (HF-FO), and HF with olanzapine and fish oil (HF-OLZ-FO). Male and female mice (*n* = 32 per sex at 4–5 weeks of age) were maintained on a high fat (HF, 48% kcal fat) diet, with fish oil, FO (D15062101; Research Diets, Inc., New Brunswick, NJ, USA) or without FO supplementation (D1562102; Research Diets, Inc.) (see Supplementary Table S1 for diet composition). In addition to the FO diets (Table S1), Olanzapine (OLZ) was administered orally after mixing with cookie dough. Animals were first given the cookie dough alone for habituation via daily exposure on Postnatal day (PND) 34, 35, and 36. From PND 37-65 (OLZ treatment period), HF and HF-FO groups received unrestricted access to their diets supplemented with daily plain cookie dough (5.89 kcal/g dough). The OLZ-supplemented groups (HF-OLZ and HF-OLZ-FO) received unrestricted access to their respective diets with daily cookie dough mixed with OLZ. To reduce risk of dough rejection, OLZ in dough was gradually reduced from 50 g/kg/day (0.06 mg OLZ/g of dough or 3 mg/kg/day) from PND 37–38 to 6.25 g/kg/day (0.48 mg OLZ/g of dough; 6 mg/kg/day) from PND 51–65. Mice were housed individually in micro isolator cages maintained at a 12 h light and dark cycle at 22 °C. Mice were euthanatized at approximately 164 days of age (range 158–170) using CO_2_. Blood and colon contents were collected from male and female mice. The colonic contents were snap-frozen in liquid nitrogen and then stored at −80 °C for further analysis. Microbial DNA was extracted from colonic contents by using the QIAamp DNA Stool Mini kit (NO. 51504, Qiagen, Germany) according to the manufacturer’s protocols. Serum and intestinal samples were collected at the end of the feeding trial for LBP and mic16S sequencing, respectively. 

### 2.2. LPS-Binding Protein Assay

LPS binding protein (LBP) was measured in serum using an ELISA kit (Hycult Inc., Wayne, PA, USA), following the manufacturer’s protocols, and as we have previously described [32].

### 2.3. Microbiome Sequencing and Analyses

DNA was extracted from each sample, and the V4 region of the 16S rRNA gene was amplified and sequenced on an Illumina HiSeq 2000 Genome Sequencer. Sequencing was performed at the Argonne National Laboratory and at the Research and Testing Laboratory (RTL), Lubbock, TX. The samples in FASTQ format were deposited to the metagenomics analysis server (MG-RAST, http://www.mg-rast.org/, accessed on 6 December 2021) in September 2018. The standard processing pipeline of MG-RAST with default settings was used for filtering sequences based on length, number of ambiguous bases, and quality. The annotation source considered was the Greengenes database using the MG-RAST processing pipeline with default settings. The bacterial community richness and bacterial community diversity indices were calculated using phyloseq R package, version 1.34.0 [33]. The observed and Shannon diversity indices were used to measure the diversity of the microbial bacteria in each group, which reflects the number of different taxa in the sample.

### 2.4. Statistical Analyses

All data were expressed with mean ± SEM (standard error of the mean). The one-way analysis of variance (ANOVA) test [34] and Student’s *t*-test [35] were used to determine the statistical significance of differences among multiple group comparison and pairwise group comparison, respectively. A *p*-value < 0.05 was considered the statistically significant difference.

## 3. Results

### 3.1. Effect of Fish Oil and Olanzapine on Final Body Weight

**Figure 1:**
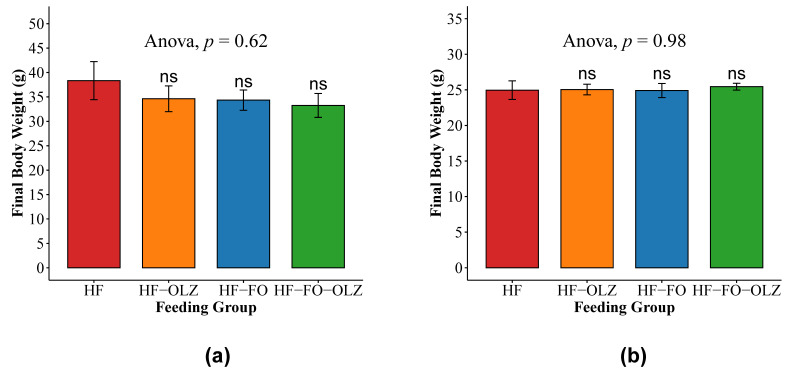
Final body weight in male (**a**) and female (**b**) mice. Data are shown as mean ± SEM for each group. ANOVA *p*-value for the multiple group comparisons are shown. The pairwise comparison is to compare the HF group versus the other groups, individually (ns for *p*-value ≥ 0.05).

Final body weight was calculated at sacrifice for the four groups in male and female mice. No significant differences in final body weights were observed between the four groups in male and female mice (Figure 1). In male mice, mice fed FO (HF-FO and HF-FO-OLZ groups) had final body weights trending lower compared to the HF and HF-OLZ groups (see Figure 1a).

### 3.2. Fish Oil Significantly Reduced Lipopolysaccharide Binding Protein (LBP) Levels in Male Mice

**Figure 2:**
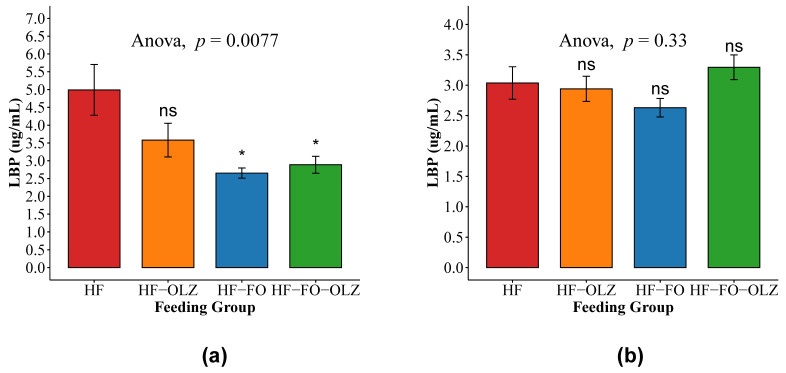
Serum lipopolysaccharide binding protein (LBP) levels in male (**a**) and female (**b**) mice. Data are shown as mean ± SEM for each group. ANOVA *p*-value for the multiple group comparisons are shown. The pairwise comparison is to compare the HF group versus the other groups, individually (* for *p*-value < 0.05, ns for *p*-value ≥ 0.05).

Serum levels of LBP in the HF group was compared to HF-OLZ, HF-FO, and HF-FO-OLZ, individually for both male and female mice. LBP levels were comparable between the HF-OLZ and HF groups, while the HF-FO and HF-FO-OLZ groups had significantly lower LBP levels compared to the HF group in male mice (Figure 2a). In female mice, there was no statistically significant difference in LBP levels across groups (Figure 2b).

### 3.3. Quality Control and Microbial Diversity

On average, 536,873 sequences were obtained from each sample. Less than 1% of sequences failed to pass the QC pipeline, 87% of sequences contained predicted features with known functions, and 13% of sequences had predicted features with unknown functions.

In male mice, alpha diversity data suggested that the richness and diversity of gut microbiota were not significantly different among the various feeding groups (*p* = 0.65 for the observed OTU index and *p* = 0.55 for the Shannon index) (see Figure S1a). In female mice, alpha diversity results indicated that the richness and diversity of gut microbiota were also not significantly different (*p* = 0.69 for the observed OTU index and *p* = 0.29 for the Shannon index) among the various groups (see Figure S1b).

### 3.4. Fish Oil Changes the Microbial Composition for Male Mice

**Figure 3:**
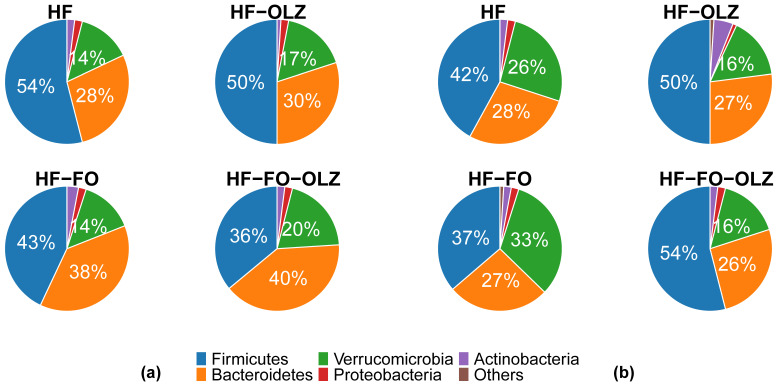
Average microbiota composition of the five highest abundance (on average with feeding group) OTUs at phylum level in the different feeding groups: (**a**) male, (**b**) female. Only major taxonomic groups are shown. Others represent the rest of the OTUs at phylum level.

**Figure 4:**
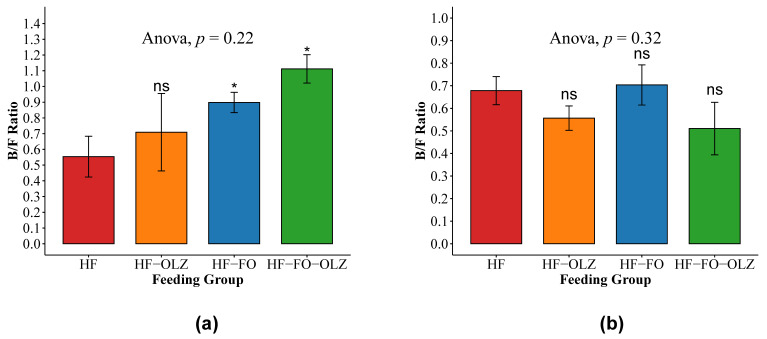
B/F ratio in male (**a**) and female (**b**) mice. Data are shown as mean ± SEM for each group. ANOVA *p* value for the multiple group comparisons are shown. The pairwise comparison is to compare the HF group versus the other groups, individually (* for *p* value < 0.05, ns for *p* >= 0.05).

In the males, the relative abundance of major microbial phyla varied between different feeding groups. *Firmicutes* was the most predominant phylum, followed by *Bacteroidetes* and *Verrucomicrobia* in HF, HF-FO, and HF-OLZ, while in HF-FO-OLZ, *Bacteroidetes* was the most predominant phylum, followed by *Firmicutes* and *Verrucomicrobia* (see Figure 3a). HF-FO and HF-FO-OLZ groups contained a higher proportion of *Bacteroidetes* and lower *Firmicutes* relative to the other groups in male mice. Bacteroidetes-to-Firmicutes (B/F) ratio was significantly increased in HF-FO (mean = 0.9) and HF-FO-OLZ (mean = 1.1) groups compared to HF (mean = 0.52) group (see Figure 4a). At all taxonomic levels, the microbial composition of mice fed FO (HF-FO and HF-FO-OLZ groups) were different from the other groups (HF and HF-OLZ) (see Figures S2–S5a and Tables S2–S6). At the class level, the Gram-negative *Bacteroidia* was substantially increased in the HF-FO and HF-FO-OLZ groups, while the Gram-positive *Clostridia* was substantially decreased compared to the HF group (see Figure S2a). At the genus level, *Barnesiella* was more enriched in HF-FO (36%) and HF-FO-OLZ (36%) relative to the HF (25%) and HF-OLZ (23%) groups (see Figure S6a).

### 3.5. Olanzapine Changes the Microbial Composition for Female Mice

In the female mice, the relative abundance of the major microbial phyla was also varied between different feeding groups. *Firmicutes* was the most predominant phylum in all the feeding groups. *Verrucomicrobia* was more enriched in the HF (26%) and HF-FO (33%) groups relative to the HF-OLZ (16%) and HF-FO-OLZ (16%) groups (see Figure 2b). The B/F ratio was not significantly different among the feeding groups (*p* = 0.18). A higher B/F ratio was observed in the HF (mean = 0.67) and HF-FO (mean = 0.69) groups relative to the HF-OLZ (mean = 0.56) and HF-FO-OLZ (mean = 0.48) groups (see Figure 4b). At all taxonomic levels, microbial composition of mice treated by OLZ (HF-OLZ and HF-FO-OLZ groups) were different from the other groups (HF and HF-FO) (see Figures S2–S5b and Tables S7–S11). At the genus level, *Akkermansia* was more enriched in HF (28%) and HF-FO (34%) relative to the other groups of HF-OLZ (17%) and HF-FO-OLZ (17%) (see Figure S6b). 

## 4. Discussion

The current study investigated changes in gut microbiota in male and female C57BL/6J mice treated in early life with SGA medication OLZ and fed an HF diet or an HF diet supplemented with FO. In contrast to the increasing body weight in adult female and male mice [36], in the current study the body weight of young female mice treated with OLZ was comparable with the body weight of HF-treated female mice, and the body weight of young male mice treated with OLZ was slightly less than the body weight of HF-treated male mice. Although OLZ failed to increase body weight in female mice, it slightly reduced body weight in male mice; the results provide an opportunity to compare changes in gut microbiota in OLZ- and HF-treated mice to compare toa previous results indicating that treatment with OLZ shifts gut bacteria toward an obesogenic profile [26].

Gut dysbiosis is an important feature of metabolic dysregulation including obesity. Dietary intervention to modulate the microbial environment is of current interest. Hence, in this study, we identified how FO along with OLZ could modulate microbes to prevent/reduce obesity. We found varied classes of microbial species to be favorably altered with FO in males, but most of the alterations were absent in female mice.

FO had a modulatory effect on the gut microbiome by increasing the B/F ratio compared to the HF mice. These data are in line with studies in rodents where obese rats had higher B/F ratio than lean mice [23]. Further, krill oil also contains PUFAS, which improved the B/F ratio similarly to our study [37]. Corroborating with that, a study of middle aged and elderly women showed that PUFA improved the microbial diversity [38]. This suggests that FO reduced the adverse effects of the HF at the microbiota level. Recent studies indicate that altered microbial composition could be used as an indicator of obesity, and the improved microbial composition due to FO consumption could be used as a simpler indicator to demonstrate its potential effects.

Obesity is associated with chronic systemic inflammation in the adipose tissue. This chronic inflammation increases the levels of circulating pro-inflammatory mediators, which could in part contribute to intestinal inflammation. Conversely, gut microbiota could be a potential source of inflammation due to higher lipopolysaccharide (LPS). In line with this, we identified that FO was able to reduce the levels of LBP protein, a marker of intestinal inflammation. FO could reduce LBP through several possible pathways. One probable pathway is through the traditional NFKB pathway. Another probability is by the metabolites of FO such as resolvins, which are known to inhibit inflammation. This needs to be further investigated but studies indicate the resolvins levels are increased with intestinal inflammation.

Acetate producing bacteria, *barnesiella* [39], was improved with the FO diet in male mice. This is important as acetate is one of the abundant short chain fatty acids (SCFA) known to prevent obesity associated inflammation in rodent models [40]. Moreover, acetate is known to improve fatty acid oxidation through increases in AMPK [41]. Furthermore, apart from its systemic role, acetate also has beneficial effects in improving insulin sensitivity in peripheral tissues [42]. In addition, the *barnesiella* was not altered in female mice, indicating the sexual dimorphic role of FO in the microbial environment. However, whether the improvements in *barnesiella* translate into increases in acetate levels remains to be tested.

Interestingly, *akkermansia Sp* is one of the few *Sp* which is intensively linked to obesity development. Lower levels of *akkermansia* are reported with obesity and insulin resistance [24]. This is important as its colonization starts in childhood and is maintained as we develop into adults. In our study, its levels were improved with FO in female mice but not in male mice. This goes hand in hand with other studies, where mice administered with *A.mucinphila* reversed the HF diet induced obesity [43,44,45]. Further, *A.mucinophila* is also known to contribute to SCFA, which in turn regulates lipid metabolism.

Furthermore, to validate the beneficial role of FO, another species, clostridia, was reduced in male mice compared to the HF fed mice that did not receive FO. This was in line with rodent studies where clostridia were higher in obese mice compared to lean mice. However, we saw the opposite results in female mice, where *clostridia* levels were higher in female FO fed mice than HF fed mice. This is in line with human studies where the *clostridia* are shown to promote weight gain in individuals exposed to *clostridia*. However, in humans several patterns emerged with obesity, due to the differences in the levels of the different species within the class [46]. It is possible that some class of *clostridia* is absent in rodents compared to humans, which still needs to be tested.

## 5. Conclusions

In conclusion, our studies showed that supplementation of HF diets with FO (HF-FO) and combined FO and olanzapine (HF-FO-OLZ) reduces dietary obesity and inflammation and induced changes to gut microbiota composition. Specifically, FO supplementation and early-life OLZ treatment increased the abundance of the genera *Firmicutes, Bacteroidetes,* and *Verrucomicrobia* in the gut microbiota and decreased the proportion of *Lactobacillus* and others, compared to HF. Additional studies are required to directly link FO and OLZ to changes in gut bacteria and subsequent reduction in obesity associated inflammation, using fecal matter transfer or treatment with the specific strains that were modified by FO and OLZ. Given that FO and OLZ are clinically used, these treatments could be repurposed for metabolic inflammatory diseases.

## Data Availability

Data supporting reported results may be requested from the corresponding author upon publication.

## Supplementary Materials

The following are available online at https://www.mdpi.com/article/10.3390/nu14020349/s1, Figure S1. Alpha diversity of both male and female mice; Figure S2. Pie chart of percent mean relative abundance of phylum level; Figure S3. Pie chart of percent mean relative abundance of class level; Figure S4. Pie chart of percent mean relative abundance of order level; Figure S5. Pie chart of percent mean relative abundance of family level; Figure S6. Pie chart of percent mean relative abundance of genus level; Table S1. Diet composition; Table S2. Phylum level relative abundance for male mice; Table S3. Class level relative abundance for male mice; Table S4. Order level relative abundance for male mice; Table S5. Family level relative abundance for male mice; Table S6. Genus level relative abundance for male mice; Table S7. Phylum level relative abundance for female mice; Table S8. Class level relative abundance for female mice; Table S9. Order level relative abundance for female mice; Table S10. Family level relative abundance for female mice; Table S11. Genus level relative abundance for female mice.
