# Supplementary material for: Sex Differences in Fish Oil and Olanzapine Effects on Gut Microbiota in Diet-Induced Obese Mice
Source: Nutrients. 2022 Jan 14;14(2):349. doi: 10.3390/nu14020349 (PMC8780445; doi:10.3390/nu14020349)
Supplement: Supplementary file 1 [file nutrients-14-00349-s001.zip › Figures_S1_S6_Supp_12012021.pdf]

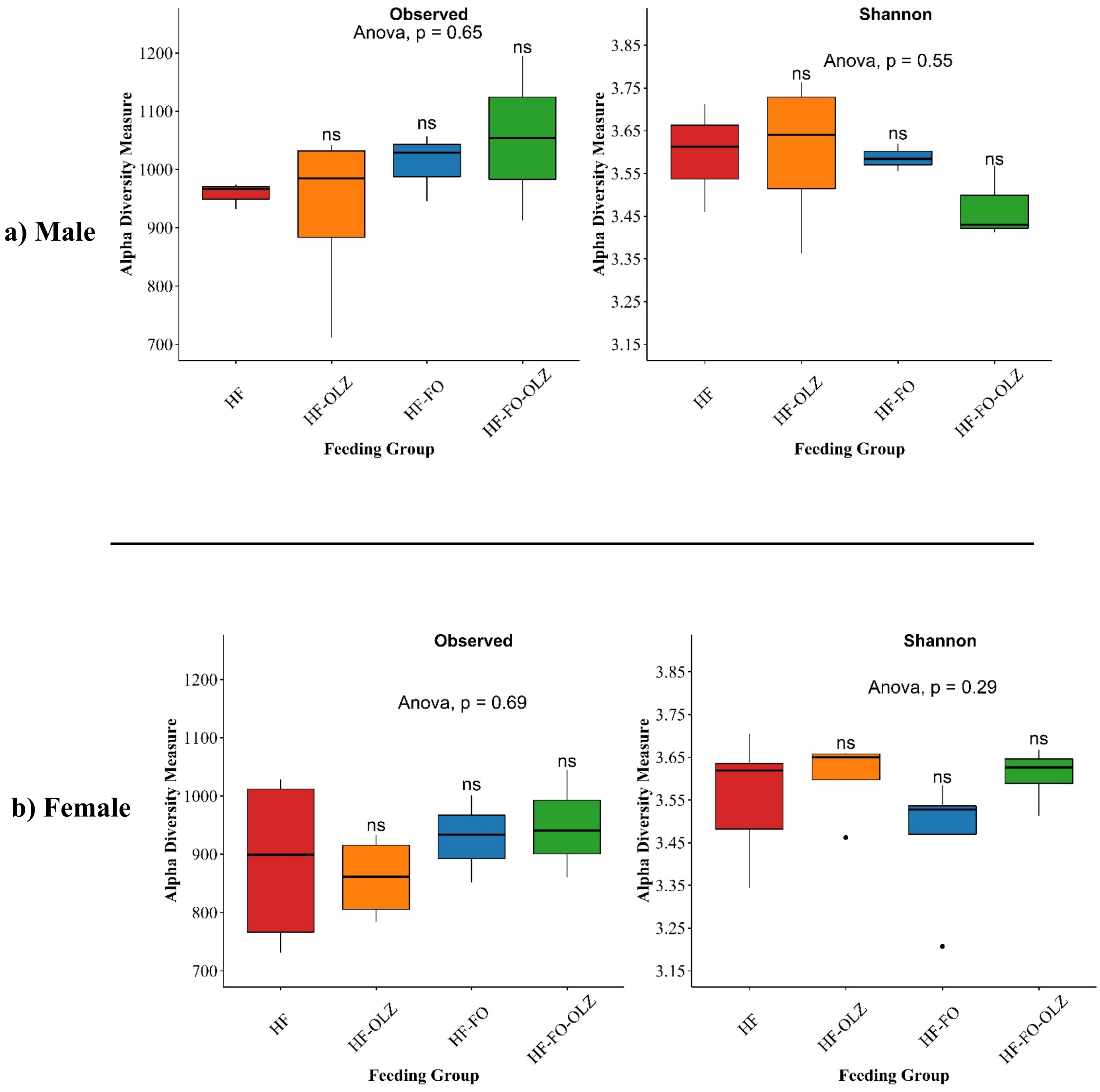

Figure S1. Alpha-diversity measured by observed species and Shannon diversity index is plotted for different feeding groups. a) Male, b) Female. The ANOVA test was used to determine the differences the significance of differences among feeding groups. The Student's t-test pairwise comparison was used to compare HF group versus the other groups, individually (ns for p >= 0.05).

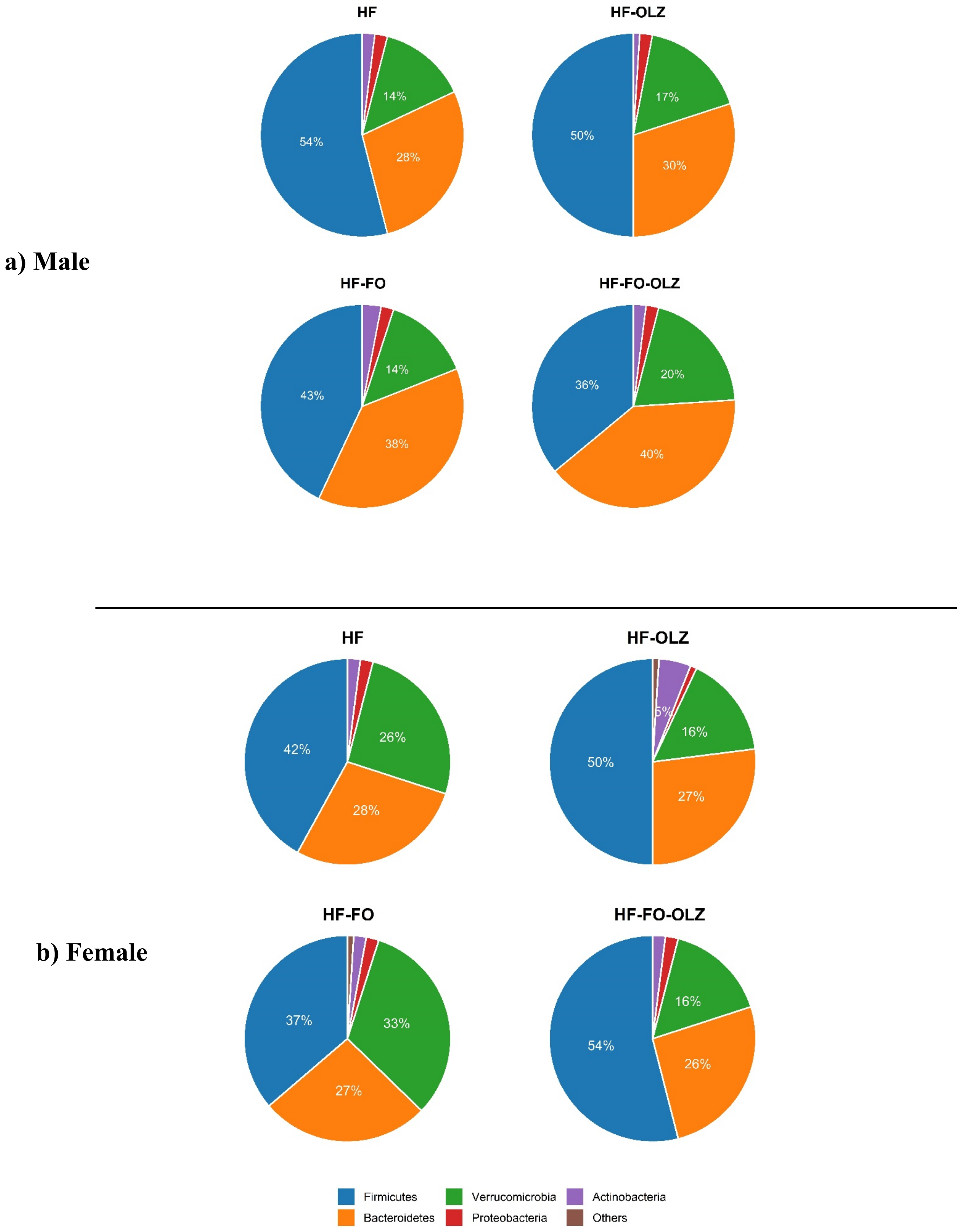

Figure S2. Average microbiota composition of the 5 highest abundance (on average with feeding group) OTUs at phylum level in the different feeding groups. a) Male, b) Female. Only major taxonomic groups are shown. Others represent the rest OTUs at phylum level.

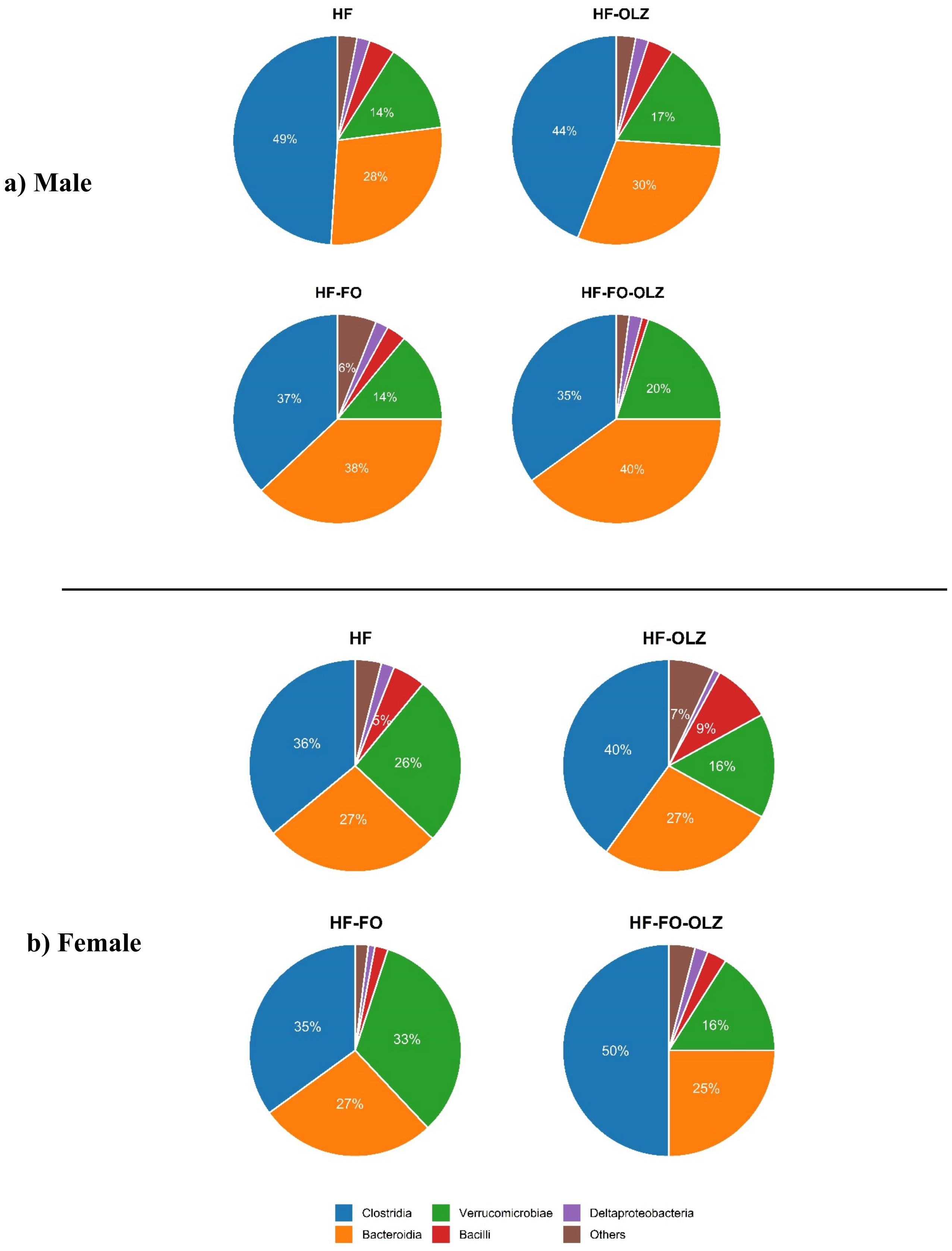

Figure S3. Average microbiota composition of the 5 highest abundance (on average with feeding group) OTUs at class level in the different feeding groups. a) Male, b) Female. Only major taxonomic groups are shown. Others represent the rest OTUs at class level.

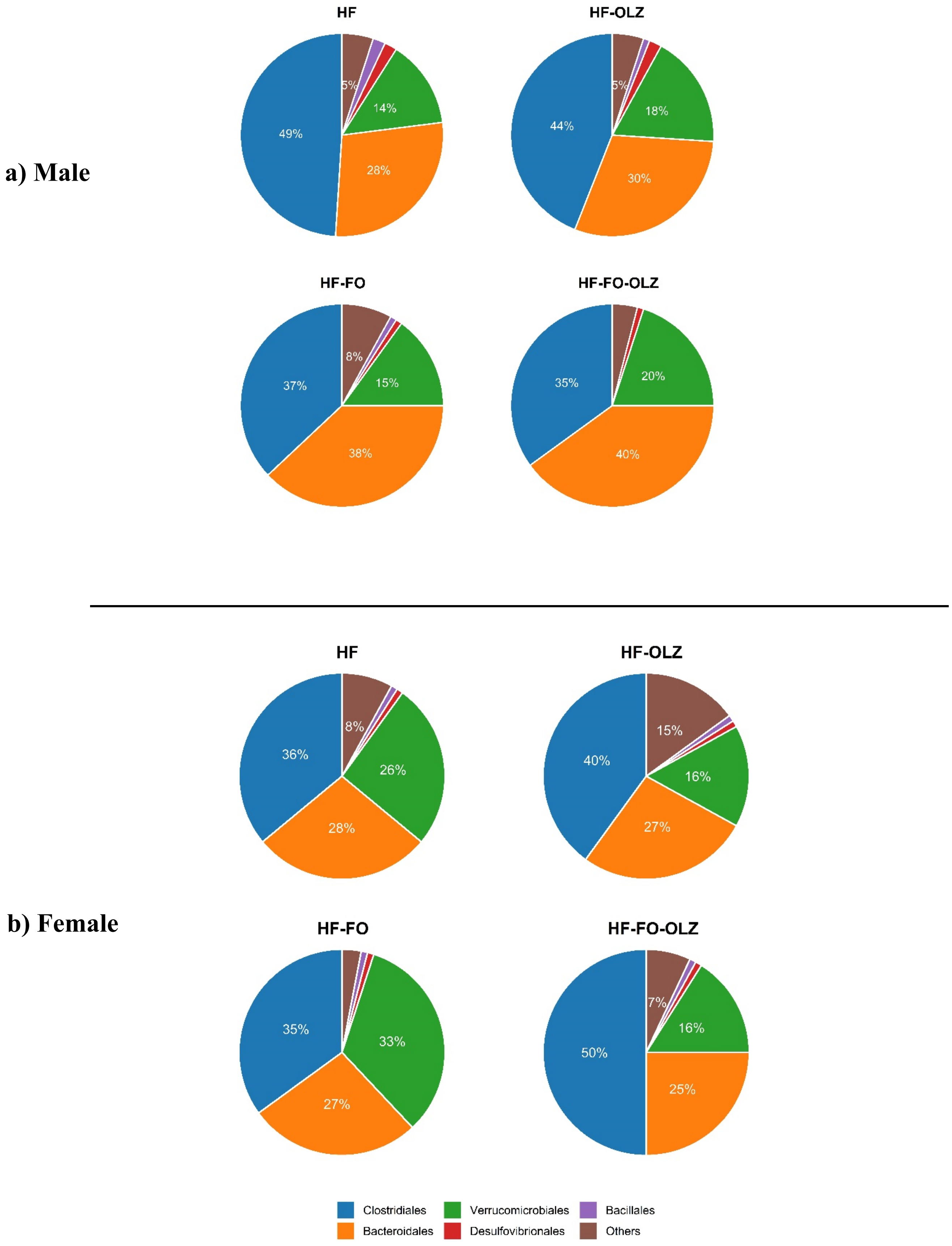

Figure S4. Average microbiota composition of the 5 highest abundance (on average with feeding group) OTUs at order level in the different feeding groups. a) Male, b) Female. Only major taxonomic groups are shown. Others represent the rest OTUs at order level.

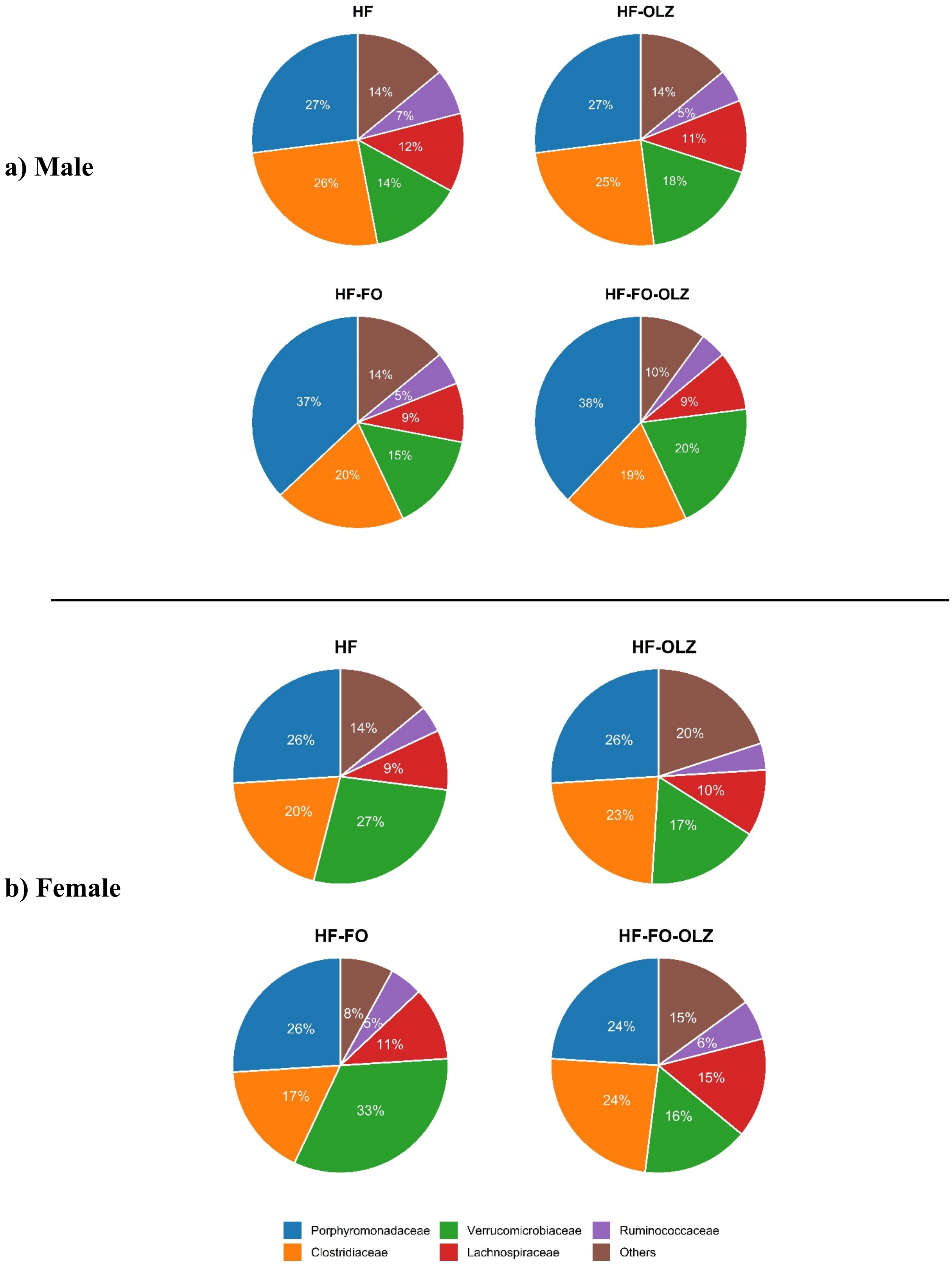

Figure S5. Average microbiota composition of the 5 highest abundance (on average with feeding group) OTUs at family level in the different feeding groups. a) Male, b) Female Colon. Only major taxonomic groups are shown. Others represent the rest OTUs at family level.

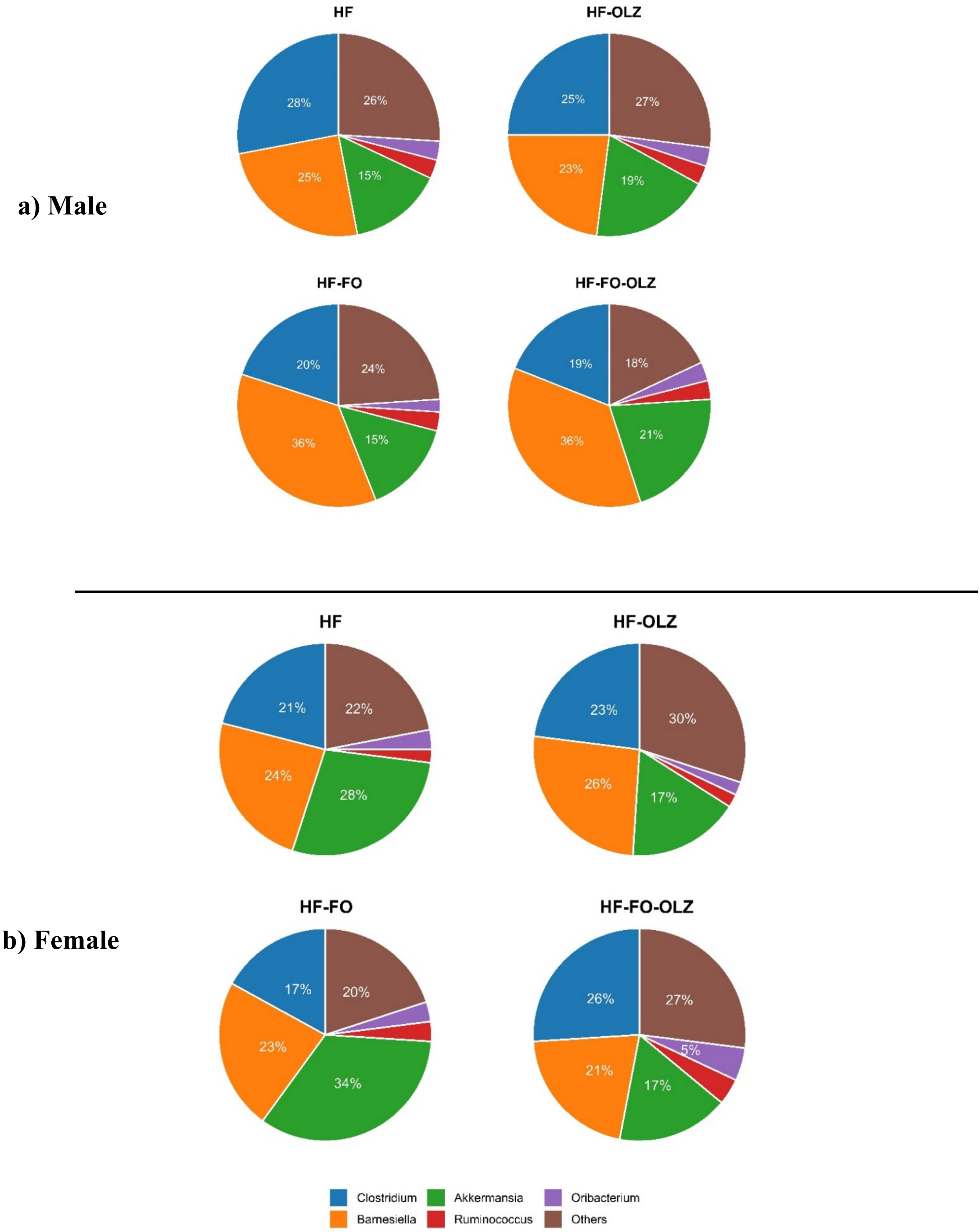

Figure S6. Average microbiota composition of the 5 highest abundance (on average with feeding group) OTUs at genus level in the different feeding groups. a) Male, b) Female Colon. Only major taxonomic groups are shown. Others represent the rest OTUs at genus level.
