# Supplementary material for: Sex Differences in Fish Oil and Olanzapine Effects on Gut Microbiota in Diet-Induced Obese Mice
Source: Nutrients. 2022 Jan 14;14(2):349. doi: 10.3390/nu14020349 (PMC8780445; doi:10.3390/nu14020349)
Supplement: Supplementary file 1 [file nutrients-14-00349-s001.zip › Table S1_Diet Composition_12012021.pdf]

Supplemental Table S1: Diet composition

	**High Fat (HF) D15062102**		**High Fat-Fish oil (HF-FO) D15062101**	
**Product #**	gm%	*kcal%*	gm%	*kcal%*
Protein	24.2	*20*	24.2	*20*
Carbohydrate	38.2	*32*	38.2	*32*
Fat	26.0	*48*	26.0	*48*
Total		*100*		*100*
kcal/gm	4.84		4.84	
**Ingredient**	**gm**	***kcal***	**gm**	***kcal***
Casein, 30 mesh	200	*800*	200	*800*
L-Cystine	3	*12*	3	*12*
Corn Starch	38	*152*	38	*152*
Maltodextrin 10	100	*400*	100	*400*
Sucrose	172.8	*691*	172.8	*691*
Cellulose	50	*0*	50	*0*
Soybean Oil	25	*225*	25	*225*
Lard	193	*1737*	144	*1296*
Menhaden Oil, Nutegrity	0	*0*	49	*441*
EPA, Ethyl Ester	0	*0*	0	*0*
Mineral Mix, S10026	10	*0*	10	*0*
DiCalcium Phosphate	13	*0*	13	*0*
Calcium Carbonate	5.5	*0*	5.5	*0*
Potassium Citrate, 1 H2O	16.5	*0*	16.5	*0*
Vitamin Mix, V10001	10	*40*	10	*40*
Choline Bitartrate	2	*0*	2	*0*
Vitamin E Acetate, 50% (500 IU/gm)	0.13	*0*	0.13	*0*
FD&C Yellow Dye #5	0.025	*0*	0	*0*
FD&C Blue Dye #1	0.025	*0*	0.05	*0*
**Total**	**838.98**	***4057***	**838.98**	***4057***
